# Preparing mental health providers for the future: the case for moving beyond the elective telehealth course to integrating telehealth training throughout the curriculum

**DOI:** 10.3389/fpsyg.2023.1301569

**Published:** 2024-01-03

**Authors:** Kathy Hsu Wibberly

**Affiliations:** Karen S. Rheuban Center for Telehealth, University of Virginia School of Medicine, Charlottesville, VA, United States

**Keywords:** training, competencies, mental health, telehealth, education, accreditation, curriculum

## Abstract

In the wake of the COVID-19 Public Health Emergency and the consequent surge in telehealth adoption, the mental healthcare landscape has undergone a profound transformation propelled by rapid technological advancements. This paradigm shift necessitates a fundamental re-evaluation of the training methodologies for mental health providers. To effectively leverage the potential of telehealth and empower mental health professionals with the requisite skills for utilizing digital tools, a comprehensive integration of telehealth into graduate program curricula is imperative. This article proposes practical strategies for seamlessly incorporating telehealth into both the theoretical and practical facets of graduate education. These strategies encompass a holistic understanding of technological platforms, ethical considerations, legal and regulatory frameworks, cultural competence, digital equity challenges, as well as clinical assessment and therapeutic approaches. The author concludes by issuing a call to action, urging mental health professions training programs and their accrediting bodies to proactively redefine their approach, ensuring mental health providers are adequately prepared for the future.

## Introduction

The progression of telehealth in mental health care delivery can be best characterized by several decades of slow and incremental growth. The onset of the COVID-19 Public Health Emergency in 2020 marked a transformative moment, sparking a rapid and unprecedented surge in telehealth adoption among both patients and providers. A study examining temporal and geographic trends in U.S. behavioral health treatment facilities (Cantor et al., 2021) revealed a 77% increase in telehealth availability for mental health treatment facilities and a remarkable 143% increase for substance use disorder treatment facilities from 2020 to 2021. In Virginia, a survey of 574 behavioral and mental health providers conducted by the Virginia Telehealth Network (2022), indicated that 97% had expanded their use of telehealth due to the pandemic, with 91% intending to maintain or further expand telehealth services. Despite this exponential growth in telehealth within the field over the past 3 years, education programs for mental health professionals persist in adhering to the traditional in-person care model. Current telemental health training, where available, is most commonly treated as a mere elective rather than an essential component of professional development.

In this article, I contend that it is essential for mental health professional training programs to transition from conventional in-person care models to hybrid care models that bring together the best of telehealth technology and digital tools with in-person care. I make the argument for why the prevailing approach of treating training in telehealth as an “elective class” is wholly inadequate for preparing the next generation of mental health providers. Finally, I extend a call to action to mental health professional training programs and their accrediting bodies, urging proactive measures to empower the next generation with the competencies required to integrate digital tools ethically and effectively into their care delivery. These include the knowledge, skills, and abilities to make sound decisions about digital tool selection, assess, and evaluate their value for improving mental health outcomes, and be better equipped to address the pressing mental health crisis facing society today and into the foreseeable future.

## A paradigm shift is needed for mental health care delivery and training

I received my Ph.D. in Counseling Psychology in 1995. On the day that I received my diploma, my formal education had provided me with no exposure to telehealth. In a chapter on the history of telehealth (Nesbitt and Katz-Bell, 2018), the 1990s were characterized as the “developmental years.” The authors highlight the launch of the American Telemedicine Association in 1993 and go on to cite multiple examples of how different states and systems began investing in telehealth, leading to the emergence of large hub-and-spoke telehealth networks.

The authors go on to describe the following decade (the 2000s) as a period of maturation for telehealth. By the end of the first decade of the new millennium, the American Telemedicine Association had published practice guidelines for videoconferencing-based telemental health (Yellowlees et al., 2010) and the American Psychological Association (APA) had published “Guidelines for the Practice of Telepsychology” (Joint Task Force for the Development of Telepsychology Guidelines for Psychologists, 2013). During this decade, I was getting established in my career in public health policy at the State level. My work was focused on enhancing healthcare access for rural and other underserved populations. In this work, I got my first glimpse at the transformative impact of telehealth on fostering meaningful and equitable access to healthcare services.

In their continuation of the history of telehealth, Nesbitt and Katz-Bell describe telehealth in the 2010s as the decade where telehealth became “mainstream” and “legitimate”. The peer-reviewed evidence supporting telemedicine and telehealth grew rapidly, encompassing over 11,000 papers identified in PubMed between 2010 and 2016. Throughout this period, the effectiveness and efficacy of telemental health in enhancing access to care became firmly established (Backhaus et al., 2012; Hilty et al., 2013). In essence, telemental health had attained the status of an Evidence-Based Practice (EBP).

In 2012, while exploring training approaches for mental health practitioners, a paper funded by the National Institute of Mental Health highlighted a lag in strategies for training in evidence-based practices (EBPs) compared to the development of the interventions themselves (Lyon et al., 2011), In that same year, I assumed my role as Director of the Mid-Atlantic Telehealth Resource Center (MATRC), one of the 12 regional and two national Telehealth Resource Centers (TRCs) funded by the U.S. Department of Health and Human Services. TRCs provide telehealth technical assistance and training to increase meaningful and equitable access to quality health care. Since access to health care, and particularly to mental health care, has been a passion and thread throughout my professional career, I thought I would reach out to my alma mater and several other graduate-level mental health provider training programs in my state to see if there might be any interest in a discussion about integrating telehealth training for the students. I even offered my professional assistance free of charge. Despite the scientific advancements, my attempts to initiate discussions about integrating telehealth training into graduate-level mental health provider programs were met with resistance. Institutions cited extensive accreditation requirements that resulted in limited room for additional content, relegating telehealth education to the status of an elective course. And since elective courses were infrequently offered and primarily dictated by student interest and demand, this stance persisted at least until 2020.

By invitation, between Summer 2020 and December 2021, I taught three graduate-level courses on telemental health best practices for two different Master's level Counseling programs and provided hundreds of hours of training for mental health professionals. By 2022, however, most graduate programs ceased offering telehealth courses, reflecting a growing chasm between education, evidence-based practices, and real-world implementation. The core curriculum and degree requirements for a Ph.D. in Counseling Psychology in 2023 remain largely unchanged from when I started my program in 1987.

In a Deloitte Insights article on the future of behavioral health (Rabinowitz et al., 2021), the authors argue that there is a clear need for private and public insurers, care providers, employers, and government policymakers to innovate to better serve the behavioral health needs of people across the world. They identified six disruption factors that they believe will drive meaningful change in the future of behavioral health. These include (1) cultural and behavioral change, (2) scientific and technological advancement, (3) increased access to care, (4) data sharing, (5) interoperable data, and (6) empowered consumers. At the core of these changes is the consumer, who will be more empowered thanks to a greater choice of behavioral health treatment and a higher quality overall experience with behavioral health care. When discussing increased access to care, the authors specifically say:

“*Emerging digital care modalities and health tools will augment in-person care, and behavioral health treatment will increasingly become a part of primary care—ensuring that scientific and technological breakthroughs are available for everyone. Already, the global healthcare mobility solutions market is expected to grow 28.4% from 2018 to 2025 to US$51B, suggesting a proliferation in direct-to-consumer behavioral healthcare solutions… Access to care is also already being streamlined by artificial intelligence that triages patients and delivers solutions customized to individual needs, such as automated reminders, and flags intervention needs in chronic disease patients. In the future, care providers will be able to integrate tools such as these and digital triage into their regular practice. Moreover, tools combined with clinical breakthroughs will allow for more effective in-patient treatment for individuals who need it.”*

A headline from MedCityNews (Carville and Mulzac, 2022) reads: “The future of mental and behavioral healthcare delivery is hybrid and choice is the key.” It was immediately followed by this tagline: “What is clear is that in-person and virtual treatment are not oppositional forces to be pitted against one another, but rather two halves of the same coin as both serve an important purpose—getting help to those who need it.” This aligns with the recognition that effectively navigating emerging digital care modalities necessitates reevaluating the foundational in-person care model.

While one of the benefits of telehealth is that it can and does increase access to care in situations where in-person care is not feasible, there is significantly more that can be done with digital health tools that augment in-person care and for which there is no in-person equivalent. Merely offering a course falls short of preparing mental health professionals for the nuanced integration of digital tools into care delivery. As the healthcare sector increasingly embraces technological innovations, including self-guided treatments, virtual assistants, augmented reality, gamified apps, digital therapeutics, wearable sensors and devices, and artificial intelligence, the need for a comprehensive overhaul in mental health training becomes imperative. Failing to adapt to these changes perpetuates a disconnect between education, evidence, and the evolving demands of real-world practice.

## Creating a core competency framework

In March 2021, the Association of American Medical Colleges (AAMC) published “Telehealth Competencies Across the Learning Continuum” as part of their New and Emerging Areas in Medicine Series (AAMC, 2021). The primary aim of these competencies is to guide educators in designing and delivering curricula, fostering the individual professional development of learners in the realm of telehealth. Notably, the AAMC explicitly states that these competencies are not designed for high-stakes assessments or accreditation purposes for schools, programs, or institutions—an assertion with which I respectfully disagree.

I contend that our foremost imperative involves establishing a robust competency framework crucial for adequately preparing mental health professionals to navigate the challenges of their evolving professional landscape, with the intention of deploying such a framework for the accreditation of schools and programs. Cavanagh et al. (2023), in their exploration of ethics and competencies essential for behavioral health professionals to seamlessly integrate digital health technologies into clinical practice, provide valuable insights. Their research methodology included a thorough literature review spanning from 2010 to 2020, extracting foundational core competencies. These competencies were then organized based on both profession and technology platform, culminating in a synthesized set of recommendations for universal and inter-professional digital competencies. Below are the six core competencies and their corresponding skills, as proposed by the authors:

- **Privacy, security, and patient safety**
  ○ Maintain privacy and confidentiality while using various digital health tools.
  ○ Understand how data transmission and security works for digital health tools and be able to communicate this clearly to patients.
  ○ Identify ways to maintain patient safety when providing behavioral health care from a distance.
  ○ Create an emergency plan with patient to address technical problems and risk management (e.g., suicide risk).
  ○ Help patient identify a support person who is located nearby that can be contacted in emergency situations and complete necessary releases of information.
- **Digital health technical skills:**
  ○ Complete training or education for relevant digital health technologies.
  ○ Be comfortable operating and instructing patients how to use digital health tool.
  ○ Be familiar with common problems for patient and provider and ways to troubleshoot them.
  ○ Understand the background and evidence-based support for digital health technologies.
  ○ Recognize when to leverage multiple digital health technologies.
  ○ Continue to expand competencies as digital tools are adapted and developed.
  ○ Identify opportunities for interdisciplinary education and teamwork on the topic of digital health.
- **Ethical and legal considerations**
  ○ Understand laws relevant to behavioral health care for provider's and patient's location.
  ○ Confirm provider meets licensure requirements for provider's and patient's location.
  ○ Obtain patient's informed consent for therapy and use digital health technologies.
  ○ Follow ethical codes and practice standards as dictated by professional association.
  ○ Set clear boundaries with patient regarding online presence and social media interactions.
  ○ Demonstrate professional behavior in person and when using digital health.
- **Clinical skills**
  ○ Confirm patient identification and location.
  ○ Establish provider identity and credibility.
  ○ Conduct an intake, gathering relevant history of patient's prior experience using digital health.
  ○ Identify appropriate assessments to administer via digital health.
  ○ Consider patient's cultural and diversity factors, including access to technology and membership to groups that may influence receptivity to digital health.
  ○ Discuss the integration of digital health into treatment plan with patient.
- **Art of therapy and digital health**
  ○ Provide clear expectations for patient-provider communication, especially when communicating over digital health (e.g., email response times).
  ○ Identify appropriate technology for patient and adjust use of digital health as needed.
  ○ Understand and inform patient of the risks and benefits associated with digital health.
  ○ Adapt therapeutic presence to foster alliance with patients while using digital health.
  ○ Monitor therapeutic alliance and identify and repair any fractures to relationship due to the use of digital health.
  ○ Reflect on digital health skills and identify areas of growth for self.
  ○ Seek consultation regarding patient care and digital health use with colleagues, supervisors, or experts as needed.
- **Administrative tasks**
  ○ Include descriptions of digital health use in clinical documentation and electronic health records.
  ○ Use billing codes to capture use of digital health technologies (e.g., telehealth) as applicable.
  ○ Identify places to efficiently integrate digital health into clinic's workflow.
  ○ Coordinate care between providers on patient's care team and inform them of the use of digital health technologies in care.
  ○ Provide outreach to community to increase knowledge of digital health tools when appropriate.

In their discussion section, the authors acknowledged the temporal scope of the reviewed articles (from 2010 to October 2020) as a limitation of their study. The dynamic landscape of telehealth, punctuated by the pandemic-fueled explosion in its adoption, has yielded a wealth of knowledge and lessons extending beyond this timeframe. For example, new insights around digital inclusion and equity underscore the necessity for providers to be able to understand how to integrate digital tools so as not to inadvertently marginalize individuals with disabilities, those facing digital literacy challenges, or those with limited access to broadband services. Recognizing the evolving landscape, there is a compelling need for continued efforts to capture these and other emergent facets of telehealth competence that have gained prominence post-2020. Consequently, I issue a call to action directed at accrediting agencies such as the American Psychological Association (APA) Commission on Accreditation, the Council on Social Work Education (CSWE), and the Council for Accreditation of Counseling and Related Programs (CACREP). This call urges these bodies to convene with experts and researchers, fostering a collaborative effort to review and enhance existing competency frameworks. A consensus should be sought on the essential elements to be standardized, forming the basis for assessing training programs. This collaborative initiative necessitates careful consideration, potentially involving the distinction between competencies that are profession-wide and those that are program or discipline-specific. For instance, PhD programs may warrant specific competencies related to telehealth research and evaluation.

## Reimagining and rebuilding the training model

Once a core competency framework has been established and built into accreditation standards, training programs will need to do the work of reimagining and rebuilding their training models to meet those standards. It should be readily apparent that the types of competencies listed in the previous section are not acquired by offering a single course on telehealth. Instead, these competencies start with the acquisition of knowledge, generally found in what are considered “core” courses. While the definition of what constitutes “core” coursework may vary across programs and disciplines, these courses, at a foundational level, provide a broad overview of human behavior. They look at the array of theories about human development and about differences, provide the historical context and evidence base surrounding different models of behavior change and behavior disorders, and explore ethical considerations relevant to professional practice and research, particularly within the context of diversity, equity, and inclusion (DEI). While much of the existing “core” course content will remain unchanged, there will be a need to integrate the history and evidence base for telehealth and related digital technologies, as well as to engage students around key considerations related to ethics and DEI within the context of telehealth. Finally, these broad overview courses will need to introduce students to the diverse array of existing and emerging digital health tools.

For clinicians in training, a requisite component of their curriculum consists of prescribed classes aimed at cultivating interpersonal and therapeutic skills. These courses, also subject to variation based on discipline and program, begin with the foundations of effective communication—honing skills in listening, reflecting, asking open-ended questions, and interpreting both verbal and non-verbal cues. As students' progress through their coursework, the curriculum delves into more advanced therapeutic skills and techniques, spanning crisis management, group processes, trauma-informed care, motivational interviewing, and a variety of therapeutic modalities. Within these classes, students actively engage in skill practice with their peers, receiving constructive feedback from both instructors and fellow trainees. While the course content will remain largely unaltered, there is a pronounced need for the integration of technology into these skill-building endeavors.

Telehealth skill building involves not only practicing therapeutic skills via technology but also receiving feedback and assessments tailored to this digital context. This integration extends to things like understanding and embodying telehealth etiquette best practices, navigating the intricacies of managing virtual group processes, and effectively engaging clients you are seeing remotely with motivational interviewing techniques. Fortunately, there is no need to reinvent the wheel as training programs can leverage existing frameworks from various contexts. Notably, the PEP Framework (Garber et al., 2023) for telehealth etiquette offers a comprehensive breakdown of the essential elements of performance, environment, and privacy/security that are crucial for successful telehealth encounters. Additionally, the iSOAP Model (Chike-Harris et al., 2021) originally designed for advanced practice registered nursing (APRN) education, proves valuable. This model serves as a guide for faculty in formulating simulation cases that authentically represent telehealth encounters and aiding them in the evaluation of students who are working to hone their telehealth skills through these simulations.

Toward the latter part of most training programs, students are required to engage in supervised clinical practice through practicums and/or internships. Since real-world practice has largely moved to a hybrid model, it would not be unreasonable to expect or even require that approved practicum and internship sites ensure that trainees have opportunities to both provide telehealth services and to engage in tele-supervision.

## Investing in the future mental health workforce

I call on mental health professional training programs and their accrediting bodies to take a proactive stance. It is imperative for the upcoming generation to be equipped with the skills to: (1) make informed and sound clinical decisions regarding the judicious use of digital tools, determining when and with whom to employ them; (2) critically assess and evaluate the efficacy of these tools in enhancing mental health outcomes, and (3) effectively address the unprecedented mental health challenges confronting our society today and into the foreseeable future.

The task of overhauling our training programs to embrace a hybrid care delivery model is undeniably challenging. It demands a dedicated commitment of time and energy, yet the resultant return on investment promises to be immensely valuable. This investment is the linchpin for empowering providers to deliver exceptional care within an increasingly digital landscape. By seamlessly integrating digital care modalities and tools into every facet of the training program, mental health professional education institutions signal their unwavering commitment to staying at the forefront of technological and scientific advancements. Moreover, they demonstrate a keen responsiveness to the evolving needs of their students and, by extension, the diverse populations these students are being prepared to serve.

## Author contributions

KW: Conceptualization, Writing – original draft.
